# Neighborhood Relationship Matters for Whom?: Interaction With Family Structure and Functional Conditions

**DOI:** 10.1093/geroni/igab046.1625

**Published:** 2021-12-17

**Authors:** Ikuko Sugawara, Erika Kobayashi

**Affiliations:** 1 Bunri University of Hospitality, Suginami-ku, Tokyo, Japan; 2 Tokyo Metropolitan Institute of Gerontology, Itabashi-ku, Tokyo, Japan

## Abstract

Living environment is considered to have unignorable effect on our health and well-being, especially when we face shrinkage of mobility as we age. Social interaction with neighbors constitutes our social environment, and it may affect our well-being by interacting with other social resources such as support from family and professional care providers. In this study we examined the effect of social environment in neighborhood on older people’s well-being, and how its effect is moderated by their family structure, functional conditions, and LTC service usage. Data was obtained from the survey conducted in 2012 with nationally representative sample of Japanese adults aged 60 years and older. The results showed that for people with functional limitation but were not certified as needing LTC, neighborhood social network was positively associated with well-being. These results suggest the unique function of neighbors for frail people to keep everyday life in the community.

